# Sorption and Desorption Isotherms of Lightweight Alkali-Activated Materials Modified with Silica Aerogel

**DOI:** 10.3390/ma18061338

**Published:** 2025-03-18

**Authors:** Halina Garbalińska, Agata Stolarska, Jarosław Strzałkowski, Agnieszka Ślosarczyk

**Affiliations:** 1 Department of Building Physics and Building Materials, Faculty of Civil and Environmental Engineering, West Pomeranian University of Technology in Szczecin, al. Piastów 17, 70-310 Szczecin, Poland; stolarska@zut.edu.pl (A.S.); jaroslaw.strzalkowski@zut.edu.pl (J.S.); 2 Division of Building Engineering and Materials, Institute of Building Engineering, Faculty of Civil and Transport Engineering, Poznan University of Technology, ul. Piotrowo 5, 60-965 Poznan, Poland; agnieszka.slosarczyk@put.poznan.pl

**Keywords:** alkali-activated materials, ground granulated blast furnace slag, fly ash-based lightweight aggregate, silica aerogel, dynamic vapor sorption

## Abstract

The moisture content in a building material has a negative impact on its technical parameters. This problem applies in particular to highly porous materials, including those based on aerogel. This paper presents moisture tests on a new generation of alkali-activated materials (AAMs) with different aerogel contents. Silica aerogel particles were used as a partial replacement for the lightweight sintered fly ash-based aggregate at levels of 10, 20, and 30 vol%. The experiment included four formulations: R0 (without the addition of aerogel) and the recipes R1, R2, and R3, with an increasing content of this additive. The level at which moisture stabilizes in a material in contact with the environment of a given humidity and temperature depends on whether the equilibrium state is reached in the process of moisture absorption by a dry material or in the process of the drying out of a wet material. The equilibrium states achieved in these processes are described by sorption and desorption isotherms, determined at a given temperature, but at different levels of relative humidity. The SSS (saturation salt solution) method has been used for years to determine them. Unfortunately, measurements carried out using this method are difficult and highly time-consuming. For this reason, a more accurate and faster DVS (dynamic vapor sorption) method was used in this study of R0–R3 composites. The research program assumed 10 step changes in humidity in the sorption processes and 10 step changes in humidity in the desorption processes. As a result, the course of the sorption and desorption isotherms of each of the four composites was accurately reproduced, and the hysteresis scale was assessed, which was most evident in the cases of the R0 composite (made without the addition of aerogel) and R1 composite (made with the lowest aerogel content). Studies have shown that the increased addition of aerogel resulted in an increase in the amount of water absorbed. This was true for all ten relative humidity levels tested. As a result, the highest values in the entire hygroscopic range were observed in the course of the sorption isotherm determined for the R3 composite with the highest aerogel content, and the lowest values were for the sorption isotherm of the R0 composite without the addition of aerogel.

## 1. Introduction

A key aspect of the drive towards a sustainable economy in the construction industry is the development of efficient, environmentally friendly building materials with a reduced embedded carbon footprint. One such solution is materials based on alkali-activated materials (AAMs). AAMs are produced by the chemical activation of waste materials, such as blast furnace slag, fly ash, ceramic waste, or glass. Activation of the source aluminosilicate materials, usually with silicate compounds, strong alkali hydroxides, or carbonates, results in a cement-like binder, but usually with increased resistance to environmental and temperature factors [1,2,3].

In view of these remarks, research into the use of AAMs in various composite solutions for mortars and concretes has accounted for a significant number of articles in recent years, highlighting the need for durability studies, including those on resistance to high temperatures and the search for new precursors and activators to reduce the carbon footprint of the final product and those addressing thermal conductivity and moisture aspects, particularly in the field of lightweight AAMs [4,5,6,7,8,9]. The latter issues are particularly relevant in the case of materials modified with ultra-lightweight nanomaterials such as silica aerogel. Silica aerogel consists of a nanoscale silica framework filled with air. This structure results in a low density (less than 0.15 g/cm^3^) with a very high specific surface area (greater than 500 [m^2^/g]) and high porosity (greater than 95%), which in turn contributes to a very low thermal conductivity (less than 0.02 [W/m·K)] [10,11]. The incorporation of silica aerogel in granular form into the structure of other materials, including lightweight cementitious or cement-like composites, has a number of desirable effects, the most important of which is a reduction in the thermal conductivity coefficient. However, due to the high porosity of silica aerogel, its incorporation into the structure of lightweight composites will also have a significant impact on the porous structure and fluid transport within the material, particularly in terms of the material’s moisture content [12,13,14,15,16].

The correct determination of the moisture content in a material is a problem of great practical importance, which is difficult to define. Nevertheless, numerous studies have been carried out in this field for many years, since the presence of water in a material has a known negative effect on most of its technical parameters.

The ability to correctly assess moisture conditions and properly control moisture transfer processes can extend the life of building components, reduce energy consumption, mitigate temperature and humidity fluctuations, and improve indoor air quality.

One of the most important properties of a material is its ability to bind moisture, which depends on the thermal and humidity conditions of the environment. The level at which the equilibrium moisture in the material will be stabilized at a given temperature and relative humidity depends on whether it is achieved in the process of sorption or desorption. Therefore, in order to ensure the full moisture characteristics of a given material, its sorption and desorption isotherms are experimentally determined. They provide information on moisture storage in building materials in the hygroscopic range, and they provide the input data used in modeling moisture transport processes.

Isotherms of sorption and desorption of porous building materials have been extensively tested around the world for years [17]. Usually, the static gravimetric method is used according to the standard in [18]. This method involves the use of desiccators to store samples at different humidity levels, stabilized by properly selected hygrostatic solutions. Studies of the kinetics of the sorption/desorption process carried out under these conditions require a lot of work, and the determination of the final sorption and desorption isotherms is difficult to obtain; this is mainly due to the long time period required to reach the state of moisture equilibrium [19].

The article in [20] discusses the advantages and disadvantages of this method and presents the results of studies that were carried out on cement pastes and concretes with the variable *w*/*c* using saturated salt solutions. Alternative techniques for measuring sorption and desorption isotherms are used to obtain this important material characteristic more efficiently. The tested sample is stored under preset environmental conditions (temperature and relative humidity) and weighed continuously. For this purpose, automated “fast” machines for dynamic water vapor sorption (DVS) [21,22,23,24] are used. While the first method is used for samples weighing tens to hundreds of grams, the second is only suitable for specimens weighing about 1000 times less, that is, from tens to hundreds of milligrams.

The DVS (dynamic vapor sorption) method was first used in the pharmaceutical, herbal, and agri-food industries. Over time, this method also began to be used for the testing of building materials, allowing faster measurements; however, the weight and volume of the samples tested were limited.

This method was successfully applied in [25] for five materials, and an experimental and theoretical analysis of water vapor adsorption in several types of porous building materials was presented.

The DVS technique was also used in [26,27] for research on earth blocks. The sorption isotherms were determined using two different methods: DVS and salt solutions. The experimental results obtained were found to be highly consistent.

In turn, in [28], the DVS technique was tested on 114 samples of unburned clay bricks and earth blocks; in [29], it was tested on natural fibers. DVS has also been successfully applied in the study of water sorption in cellulosic materials [30]. The water vapor sorption properties of spruce were studied in [31] using a dynamic steam sorption apparatus.

The hygrothermal properties of unfired earth bricks with the addition of barley straw, hemp shives, and corn cobs were tested in [32], using the DVS method and the saturated salt solution method. In the study in [33], the hygrothermal properties of five earth bricks were measured using two methods, including the DVS method.

The DVS method is widely used to test cement-based materials [23,34,35,36,37,38,39,40,41,42].

The article in [43] presents comprehensive studies carried out for hardened cement pastes. Dynamic water vapor sorption experiments were performed using the DVS-Adventure analyzer. The equivalence of the DVS method and the conventional saturated salt method was demonstrated and confirmed.

In [44], prefabricated hemp concrete was investigated. Sorption isotherms were determined using various test methods: saturation salt solution (SSS) methods with a single relative humidity step or stepwise equilibrium and dynamic vapor sorption (DVS).

A comparison of both the SSS and DVS methods was also made in [19,45], where aerated concrete of four density classes was tested in an extensive experiment. Independent comparative studies on desorption isotherms, presented in [45], and comparative studies on sorption isotherms of all the studied classes, described in detail in [19], were carried out. Both the sorption and the desorption isotherms obtained with the application of the two methods showed good compatibility in all of the concrete tested, within the humidity range from 0% to 75–85%. In the high humidity range, from 75–85% up to 98%, there appeared to be a considerable diversification of sorption and desorption isotherm courses determined by the two different methods; this was particularly observed in the case of the higher density classes.

In the present work, taking into account the above-mentioned advantages and the relatively short measurement time of the DVS method compared to the SSS method, it was decided to use this technique to determine the sorption and desorption isotherms of the tested alkali-activated lightweight materials modified with different amounts of silica aerogel (R0, R1, R2, and R3). After the first extensive phase of the research, which demonstrated the favorable physical, mechanical, and thermal parameters of the tested AAMs (see [6]), the research team undertook to prepare the moisture characteristics for all four composites in the form of sorption and desorption isotherms.

In the previous stage, only the basic moisture parameter, i.e., water absorption, was determined. The scientific novelty of the presented work is the determination of the sorption and desorption properties of the AAM composites containing aerogel granules, which are missing in the available scientific literature. This paper presents research dedicated to the detailed determination of the equilibrium moisture content at ten RH levels, covering the entire hygroscopic range. Equilibrium values were determined for both the sorption and desorption processes, taking into account the occurrence of hysteresis in this type of material. Based on previous experience and literature reports, care was taken to ensure proper experimental preparation, including adequate drying of the samples and appropriate programming of the DVS IntrinsincPLUS instrument.

## 2. Materials and Methods

An extensive research program was carried out for four alkali-activated composites R0, R1, R2, and R3, which differed in aerogel content, with the R0 recipe being the reference formulation made without the addition of aerogel.

To prepare the lightweight composites R0–R3, ground granulated blast furnace slag GGBFS and a mixture of sodium silicate and sodium hydroxide at a concentration of 3 M were used. Ground granulated blast furnace slag, obtained as a by-product of steelmaking, with a high vitreous phase content of more than 95% and the following oxide composition in %: CaO—39.7, SiO_2_—38.2, Al_2_O_3_—10.2, MgO—7.8, SO_3_—1.6, Fe_2_O_3_—1.3, K_2_O—0.8, Na_2_O—0.3, and P_2_O_5_—0.1, was used as a binder source. An artificial aggregate obtained from fly ash, with a grain size of 1 to 4 mm, characterized by an irregular shape, a low coefficient of thermal conductivity 0.16 [W/(m·K)], and a low bulk density of 620 [kg/m^3^], was used as the lightweight aggregate for producing the AAMs. Silica aerogel granules with a particle size range of 1.2 to 4 mm and a high porosity exceeding 90% were also used as a partial replacement of the aggregate. According to the manufacturer, the silica aerogel is characterized by low bulk density of 65–85 [kg/m^3^], low thermal conductivity of 0.012 [W/(m·K)], temperature stability up to 600 °C, and hydrophobic surface properties.

The following quantities of raw materials were used to prepare the base recipe R0: 750 [kg/m^3^] GGBFS, 534.4 [kg/m^3^] waterglass, 128.1 [kg/m^3^] sodium hydroxide, and 600 [kg/m^3^] fly ash-based lightweight aggregate. Recipes R1, R2, and R3 had the same composition as the base formulation with some of the lightweight aggregate replaced by silica aerogel at 8.9, 17.8, and 29.6 [kg/m^3^], respectively. All the components of the mixtures were mixed in a Hobart mixer until a homogeneous composition was obtained; then, prismatic specimens of 4 × 4 × 16 cm were formed for the physical and mechanical tests, and cubic specimens with dimensions of 10 × 10 × 10 cm were formed for the hygrothermal tests. After demolding, the specimens were conditioned in water at an elevated temperature of 80 °C for 28 days.

A very extensive multi-stage experiment was carried out to diagnose the properties of these composites made from alkali-activated blast furnace slag, fly ash-based lightweight aggregate, and silica aerogel added in varying amounts.

The paper in [6] shows the basic physical and mechanical properties of the lightweight composites R0, R1, R2, and R3. In the paper in [6], the following properties of the tested composites (R0–R3) were presented and analyzed: flowability, density, water absorption, thermal conductivity, volumetric specific heat, thermal diffusivity, flexural strength, and compressive strength.

This article is a continuation of the previous research, and its aim is to examine the hygroscopic properties of the four analyzed materials—in particular, it aims to determine their sorption and desorption isotherms.

Measurements of the hygroscopic sorption properties of building materials and products are described in the EN ISO 12571 standard [18]. This standard specifies two alternative methods:

(a) Using desiccators and weighing cups (desiccator method);
(b) Using a climatic chamber (climatic chamber method).

The desiccator method is recognized in [18] as the reference method. With this method, saturated aqueous solutions of appropriate salts are prepared to achieve the required relative humidity in the individual desiccators. Then, the desiccators with selected solutions and tested specimens are placed in the constant-temperature chamber. For both sorption and desorption, at least four test atmospheres should be selected.

Before starting sorption measurements, samples should be dried to a constant mass. Equilibrium with the environment is established by weighing the specimens until constant mass is reached. At this point, a uniform moisture distribution in the samples is assumed. After establishing the equilibrium moisture content at each relative humidity, the sorption curve of the test material can be drawn. In a similar way, research is carried out to determine desorption isotherms. The starting point for desorption is a relative humidity of at least 95%. While maintaining a constant temperature, the specimens are placed consecutively in a series of test environments, with relative humidity decreasing in stages. Finally, the specimens are dried to a constant mass. On the basis of the equilibrium moisture content determined in each climate, a desorption isotherm can be prepared for the tested material.

The use of the desiccator method to determine sorption and desorption isotherms requires a lot of work and many months of systematic measurements of the changing mass of individual samples in each climate.

Determination of hygroscopic sorption/desorption properties is much easier and faster with the DVS method. The dynamic vapor sorption (DVS) technique is a highly sensitive, accurate, and rapid means of obtaining an automated determination of the moisture sorption/desorption properties of solids. The experimental set-up consists of a sensitive balance inside a well-controlled environmental chamber. The weight of the sample is measured as a function of time as it absorbs moisture from the atmosphere (sorption) or releases moisture into the atmosphere (desorption).

The dynamic vapor sorption (DVS) analyzer used in this work was the DVS IntrinsicPLUS (from Surface Measurement Systems Ltd., London, UK), which can operate up to 40 °C and covers the 0–98% range of RH.

An undoubted advantage of DVS is that the measurements are actually carried out under isothermal conditions, by directly controlling and changing relative humidity, instead of reconstructing isotherms from isobaric conditions in desiccators [46].

Another advantage of DVS is that both relative humidity and temperature are controlled with high precision, whereas such accurate maintenance is much more difficult using the conventional desiccator method [46].

The DVS IntrinsicPLUS has temperature control to ±0.1 °C, ensuring excellent instrument baseline stability as well as accurate control of relative humidity generation to ±1% RH [47].

Samples were prepared for the DVS tests in a dry state. Therefore, before the start of the main measurements, the samples of each composite (R0–R3) were kept in a dryer at 105 °C until they were completely dried, i.e., a constant mass was obtained for each recipe.

**Figure 1:**
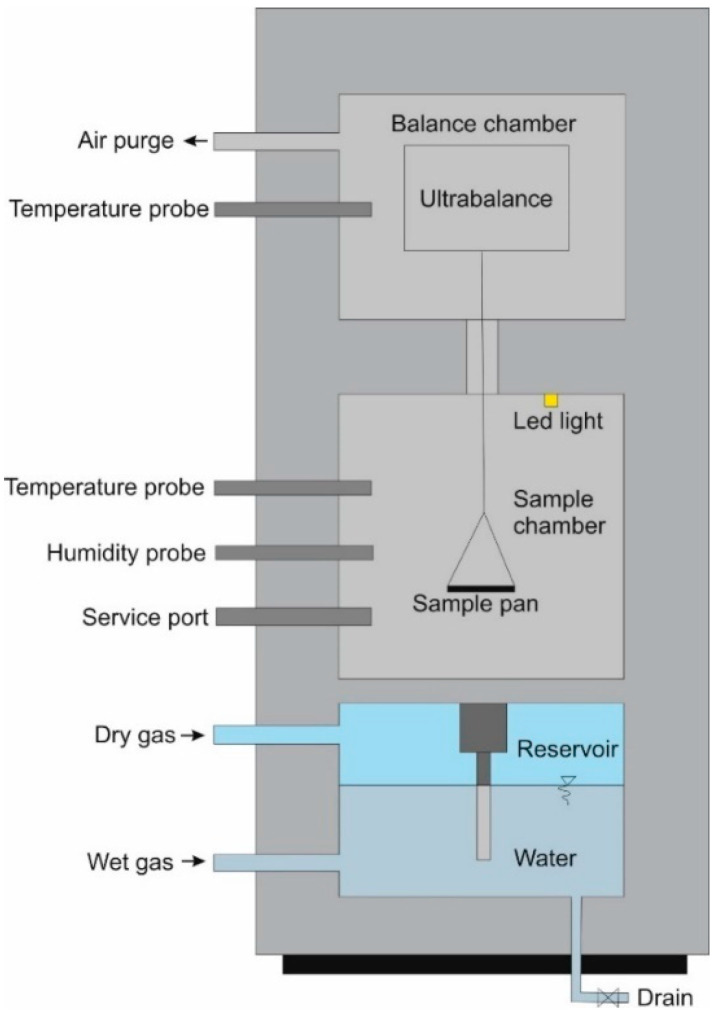
Schematic of the main components of the DVS IntrinsicPLUS, based on [47].

**Figure 2:**
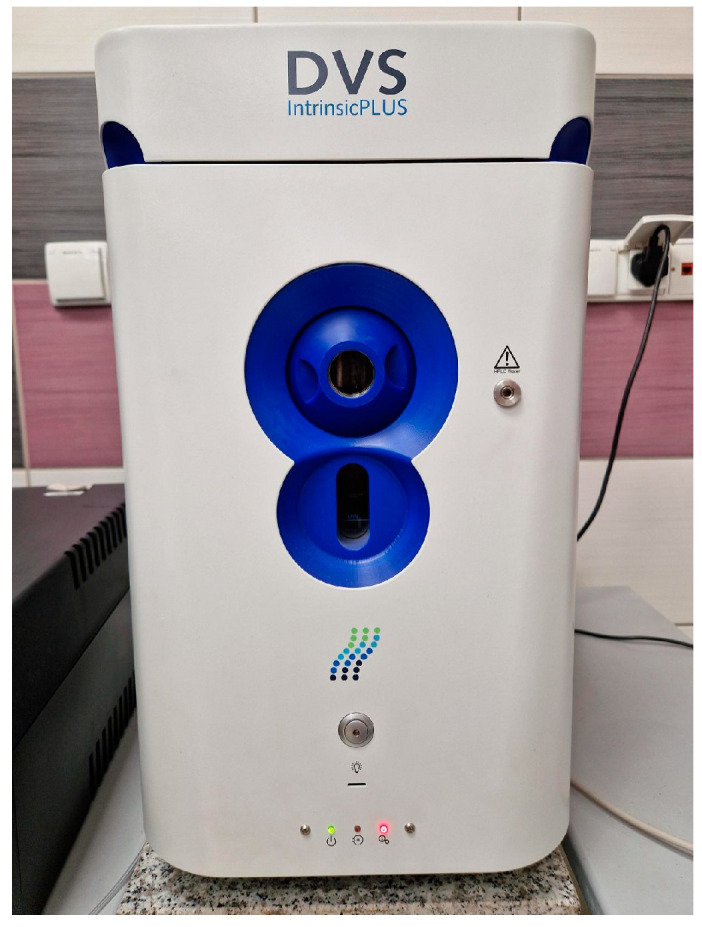
View of the DVS IntrinsicPLUS instrument used in the study.

A schematic diagram of the DVS instrument is shown in Figure 1, while Figure 2 shows a photo of the DVS IntrinsicPLUS instrument used in the study.

The DVS IntrinsicPLUS is fully automated, under the control of the DVS-WIN software package (DVS Control Software version 1.3.3.0) supplied with the instrument, providing a flexible and easy-to-use interface for setting up and running moisture sorption/desorption experiments [47].

This software allows automatic transition to subsequent adsorption and desorption cycles without the need to remove samples, unlike desiccator testing, where the samples are often transferred from one chamber to another or the chamber is opened to exchange salt solutions or to weigh the samples [46].

In order to determine the moment of reaching the moisture equilibrium, the device systematically records changes in mass over time (d*m*/d*t*). When the d*m*/d*t* value is close to 0, the next humidity level is set automatically. In the tests carried out, an air temperature of 20 °C and a range of relative humidity of 0–97%, with a change of ≈10%, were selected as test conditions. Each time the control program detected a mass change of less than 0.0005% per minute, the relative humidity was automatically changed by approximately 10%.

## 3. Results and Discussion

**Figure 3:**
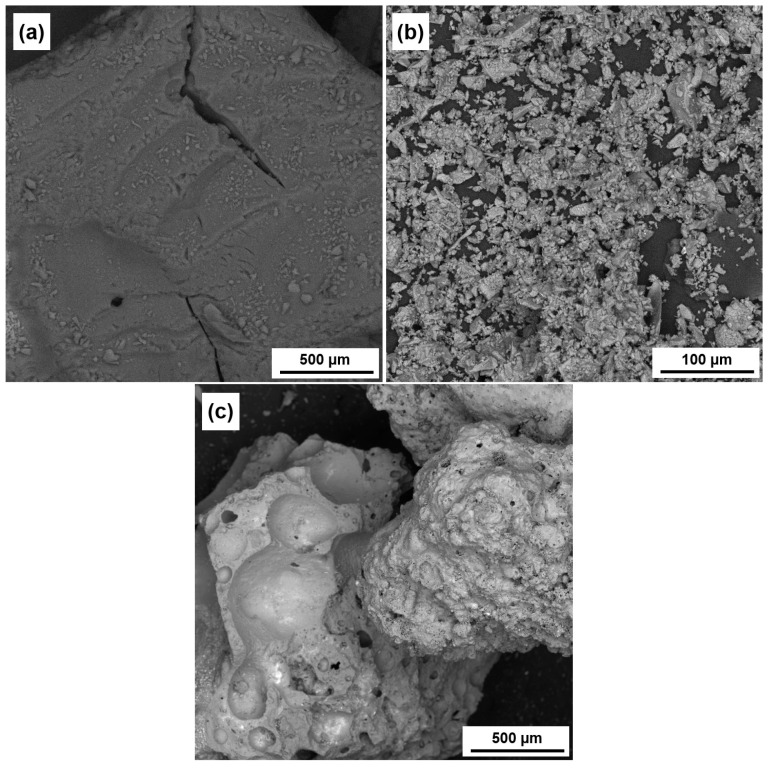
The images of the microstructures of the materials used: (**a**) silica aerogel, (**b**) GGBFS, (**c**) fly ash-based aggregate.

Figure 3 shows scanning electron micrographs of the source materials: silica aerogel, granulated blast furnace slag and fly ash-based aggregate; the point analysis of the elemental compositions of the aforementioned materials is summarized in Table 1. SEM images and EDS analysis were performed using a Tescan 3Vega scanning microscope (Tescan Group, Brno, Czech Republic) equipped with a Bruker microprobe (Bruker, Karlsruhe, Germany).

**Figure 4:**
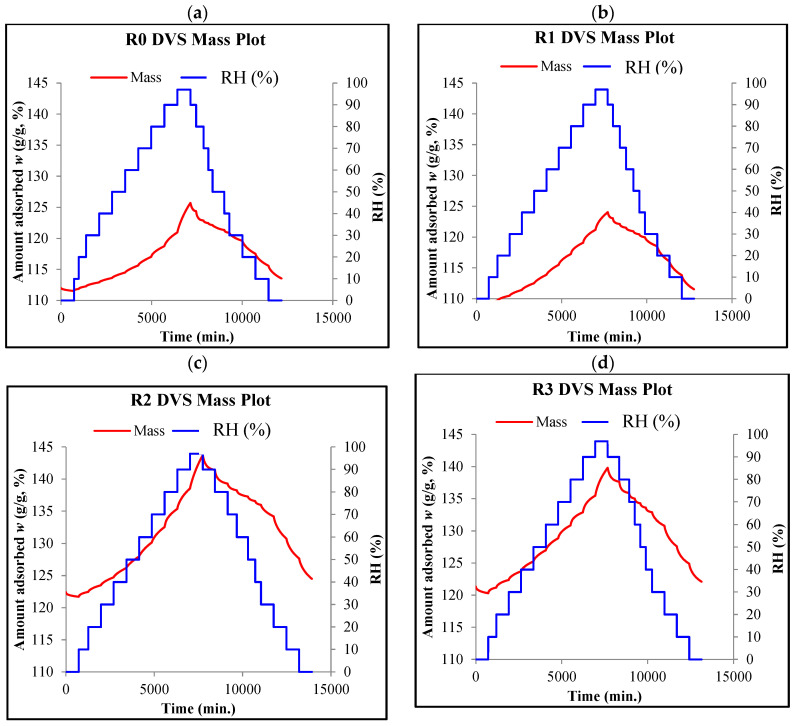
Diagrams showing the courses of DVS measurements for the tested composites: (**a**) R0, (**b**) R1, (**c**) R2, (**d**) R3.

Figure 4 presents the diagrams (obtained in the DVS tests) showing the course of the sorption and desorption measurements for the tested R0, R1, R2, and R3 composites.

Table 2 presents the equilibrium moisture values *w* [%] that were determined for the R0–R3 composites in the sorption studies carried out using the DVS technique under gradually changed humidity conditions. For each of the materials, changes in humidity from a minimum level of 0% to a maximum level of 95% were programmed, assuming a step increase in humidity in the chamber in the range of 0–90% in 10% increments.

Table 2 shows the values of stabilized moisture *w* [%] for each of the composites, but they are assigned to the real humidity levels actually occurring in the sample chamber in the subsequent stages of the sorption processes.

Table 3 presents analogous data for the R0–R3 composites but refers to the desorption processes carried out using the DVS technique.

**Figure 5:**
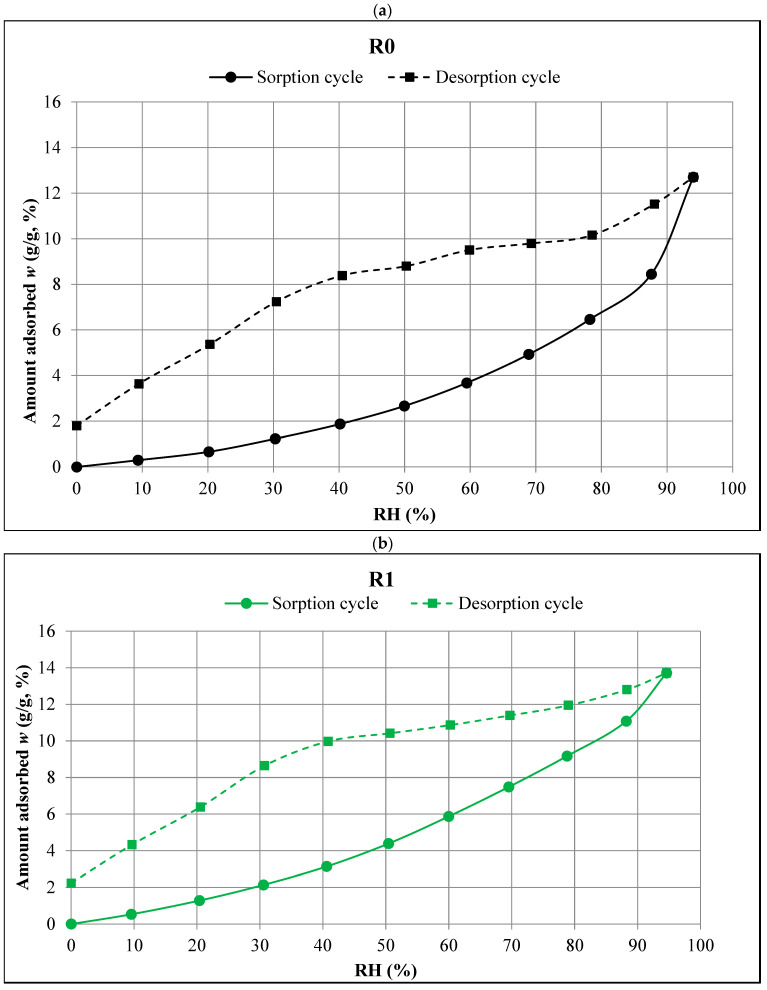

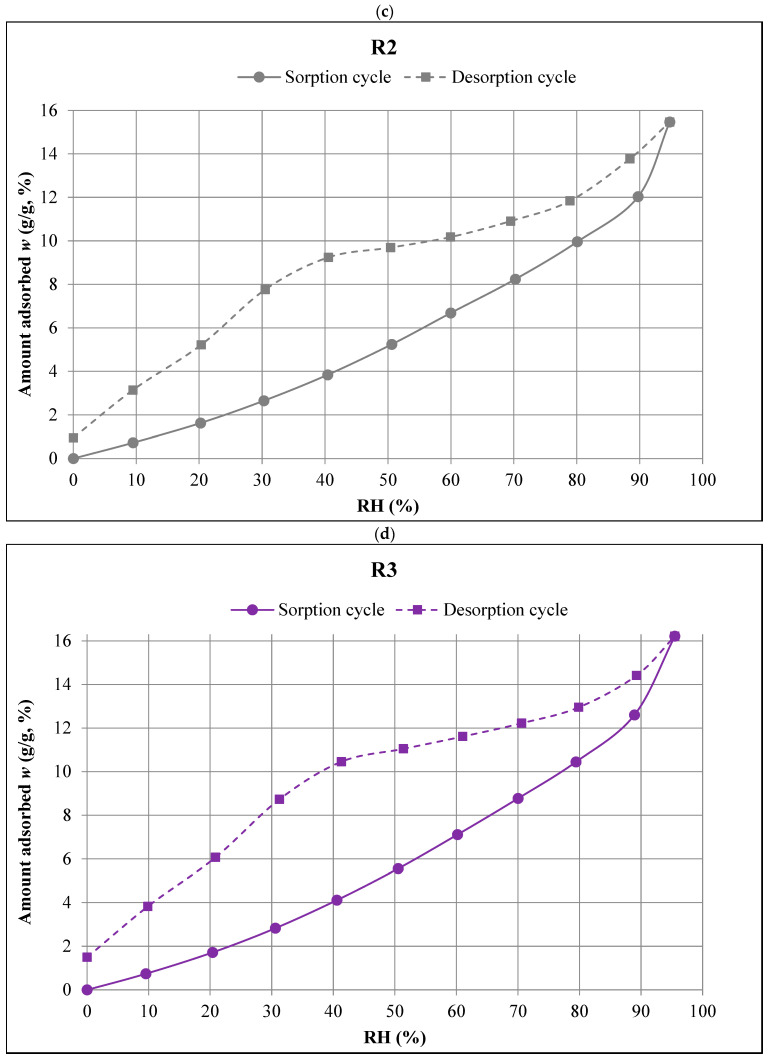
Sorption and desorption isotherms of the tested composites: (**a**) R0, (**b**) R1, (**c**) R2, (**d**) R3.

**Figure 6:**
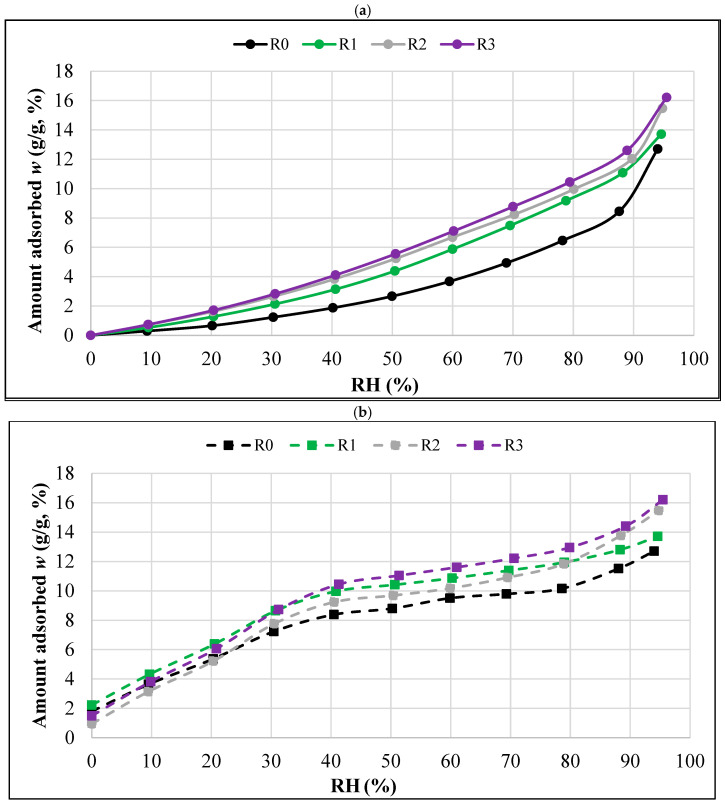
Sorption (**a**) and desorption (**b**) isotherms of the tested composites.

**Figure 7:**
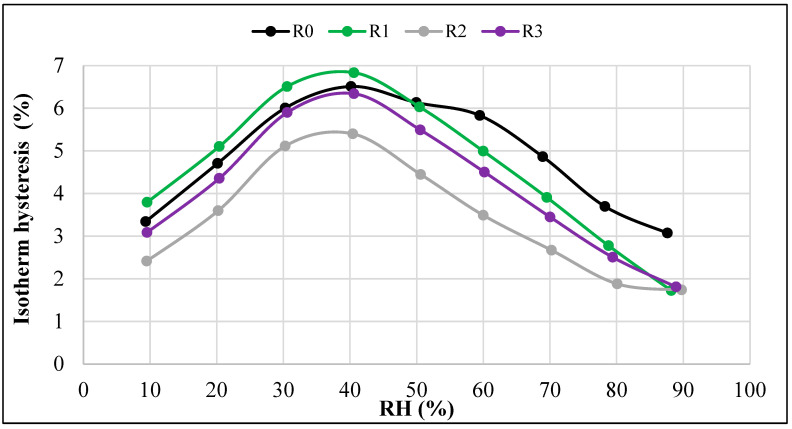
Isotherm hysteresis of the tested composites.

The obtained RH levels differed slightly from the values programmed in the DVS IntrinsicPLUS instrument used in the study. In most cases, these differences did not exceed 1%. It should be emphasized, however, that in Figure 5, Figure 6 and Figure 7 (prepared on the basis of Table 2 and Table 3) there are RH values actually occurring in the test chamber of the DVS apparatus.

The results obtained in the DVS measurements confirm the dependence of the increase in equilibrium moisture on the increase in relative humidity. The highest sorption moisture content was obtained for all the samples at the highest RH level, and in the extreme case, i.e., for the R3 formula, it was about 16.2%. On the other hand, the lowest sorption moisture content with the highest RH was recorded for the R0 formulation, and it was at the level of approximately 12.7%.

The data collected in the sorption (Table 2) and desorption (Table 3) processes are graphically illustrated in Figure 5a–d, which show the sorption and desorption isotherms for each of the R0–R3 composites studied.

The values in Table 2, obtained using the DVS technique in the sorption processes, were used to prepare the summary plots presented in Figure 6a. This comparison allows the courses of the sorption isotherms of each of the tested composites (R0, R1, R2, R3) to be compared.

Both the non-aerogel composite (R0) and all the composites with different aerogel additions (R1, R2, R3) obtained type III isotherms according to the Brunauer and IUPAC classifications. The shape of a type III isotherm is concave with respect to the RH axis throughout the range and has no inflection point.

The highest absorption capacity in each RH humidity range was found in the R3 composite with the highest aerogel content. By far the lowest equilibrium moisture content was shown by the R0 composite without the addition of aerogel.

Figure 6b was prepared in an analogous manner to Figure 6a. Figure 6b presents a summary diagram, prepared on the basis of Table 3 and showing the desorption isotherms of all the tested composites.

Figure 7 illustrates the differences in equilibrium moisture stabilized (at analogous RH levels) in the tested composites as a result of the sorption and desorption processes. This occurs when the amount of moisture adsorbed under given conditions during the sorption process is lower than the amount of moisture stabilized as a result of the desorption process. Hysteresis is often temperature-dependent and can be caused by various factors, as explained in the study [35].

Analyzing the data presented in Figure 7, it should be stated that the differences between the equilibrium moisture values obtained in the sorption and desorption processes depend on the humidity level. The largest differences occur in the intermediate RH ranges, and the smallest in the lowest and highest ambient humidity.

## 4. Conclusions

Based on the results obtained, the following conclusions can be formulated:

- The DVS method provided an effective determination of the equilibrium moisture values (*w*) for all the tested composites at each humidity level (RH).
- For each composite (R0–R3), ten measurement points were obtained in each sorption cycle and each desorption cycle, which provided the possibility of a precise determination of the course of both the sorption isotherms and desorption isotherms.
- All the tested composites exhibited Type III isotherms according to the Brunauer and IUPAC classifications. This type of isotherm occurs when the interactions between the adsorbate molecules are much stronger than those between the adsorbate and the adsorbent. Capillary condensation occurs in the pores of the adsorbent.
- The R0 composite, which did not contain aerogel, had the lowest sorption moisture values *w* in the entire humidity range tested (0–95%). Increasing the aerogel content led to higher moisture absorption. The R3 composite, with the highest aerogel content, showed the highest values of equilibrium sorption moisture *w* at all RH levels.
- In the range of the highest relative humidity (RH > 90%), the influence of capillary condensation is clearly visible, leading to an intense increase in the equilibrium sorption moisture *w*. It is most significant in the case of the R0 composite, with the most compact porosity structure.
- Capillary condensation also affected the course of the desorption isotherms of all the composites, leading to the more intense increase in the equilibrium values of *w* in the range RH > 80%.
- The desorption trends were similar to the sorption trends at RH > 30%, i.e., the lowest equilibrium values of desorption moisture *w* were found in the case of the R0 composite and the highest were in the case of the R3 composite.
- In the range of the lowest humidity (RH < 30%), no clear differences were observed between the desorption isotherms of the individual composites. However, in all the composites, there was a significant decrease in equilibrium moisture values *w* with a decrease in humidity. The changes in this range (RH = 30% → 0%) were much greater than those recorded in the measurements of the sorption isotherms.
- The differences between the sorption and desorption equilibrium moisture *w* depended on the RH values. The largest differences, ranging from 5% to 7%, occurred at intermediate RH levels. The smallest differences were observed at both the lowest and highest RH levels.
- The hysteresis was most pronounced in the R0 and R1 composites, i.e., those made without the addition of aerogel or with the lowest aerogel content. In the R0 composite, the hysteresis was more pronounced at RH > 50%, and in the R1 composite, the hysteresis was more evident at RH < 50%.

The collected data on the sorption and desorption properties in the whole range of hygroscopic humidity allow the determined sorption and desorption isotherms to be linked with other technical parameters, which are most often strongly dependent on moisture content.

By ensuring the proper moisture content, the alkali-activated composites with aerogel additives offer excellent thermal insulation, fire resistance, and moisture regulation, making them ideal for energy-efficient construction and fireproof coatings. Their lightweight nature allows them to be used in prefabricated elements, while their high porosity helps to regulate indoor humidity. Additionally, they contribute to sustainability by utilizing industrial by-products and reducing CO₂ emissions.

The paper in [6] presents the stage of earlier research on the basic physical and mechanical properties of lightweight composites (R0–R3): flowability, density, water absorption, thermal conductivity, volumetric specific heat, thermal diffusivity, flexural strength and compressive strength. This article is a continuation of the previous research, and its aim is to examine the hygroscopic properties of tested materials—in particular, the aim is to determine their sorption and desorption isotherms.

Further research is planned and will be carried out in order to diagnose the impact of the moisture level on the basic thermal and strength properties, i.e., to reproduce the variability of their values in accordance with the determined sorption and desorption isotherms of the tested composites. These studies will determine the practical use of R0-R3 composites in relation to the expected conditions of their application.

Complementary research is also planned, including research on the hygroscopic properties of the raw materials, including the aerogel itself—using the DVS technique and analogous RH settings, as in the R0—R3 tests.

Due to the limitations of the DVS method, which are mainly related to the weight of the tested samples, the authors also plan to continue the research on the isotherms of individual R0–R3 composites using the traditional SSS (saturation salt solution) method; then, after their completion, appropriate comparisons will be made—in a manner similar to that which was conducted in the article [19].

## Figures and Tables

**Table 1:** The elemental analysis of raw materials.

Chemical Element Content, wt.% by EDS Analysis					
Silica Aerogel		GGBFS		Fly Ash-Based Aggregate	
Oxygen	52.27	Oxygen	31.96	Oxygen	52.06
Silicon	36.48	Silicon	11.40	Silicon	17.53
Carbon	11.25	Carbon	10.01	Carbon	6.54
		Calcium	37.35	Calcium	8.44
		Aluminum	3.07	Aluminum	6.95
		Potassium	0.66	Potassium	3.26
		Magnesium	2.85	Magnesium	1.56
		Iron	1.21	Iron	1.53
		Sulfur	0.60	Sodium	1.38
				Titanium	0.75

**Table 2:** Equilibrium moisture contents determined in the DVS sorption experiment for the composites R0–R3.

Measured RH [%]	R0 *w* [%]	Measured RH [%]	R1 *w* [%]	Measured RH [%]	R2 *w* [%]	Measured RH [%]	R3 *w* [%]
0.00	0.000	0.00	0.000	0.00	0.000	0.0	0.000
9.34	0.295	9.56	0.532	9.49	0.726	9.5	0.737
20.14	0.663	20.38	1.281	20.21	1.626	20.4	1.714
30.27	1.234	30.55	2.136	30.28	2.649	30.6	2.828
40.15	1.876	40.59	3.140	40.41	3.840	40.6	4.113
49.95	2.666	50.43	4.390	50.60	5.243	50.5	5.556
59.47	3.677	59.98	5.871	59.98	6.686	60.2	7.111
68.91	4.931	69.52	7.484	70.23	8.239	70.0	8.772
78.23	6.463	78.78	9.170	80.08	9.959	79.4	10.445
87.61	8.448	88.19	11.079	89.74	12.033	88.9	12.600
94.00	12.703	94.59	13.712	94.77	15.470	95.5	16.211

**Table 3:** Equilibrium moisture contents determined in the DVS desorption experiment for the composites R0–R3.

Measured RH [%]	R0 *w* [%]	Measured RH [%]	R1 *w* [%]	Measured RH [%]	R2 *w* [%]	Measured RH [%]	R3 *w* [%]
0.00	1.798	0.00	2.228	0.00	0.948	0.0	1.492
9.49	3.638	9.66	4.327	9.47	3.141	9.9	3.824
20.30	5.370	20.53	6.385	20.33	5.223	20.9	6.070
30.44	7.238	30.72	8.644	30.49	7.762	31.2	8.728
40.48	8.387	40.84	9.974	40.53	9.240	41.3	10.454
50.24	8.800	50.65	10.420	50.40	9.691	51.4	11.048
59.87	9.507	60.22	10.866	59.93	10.177	61.0	11.611
69.31	9.794	69.71	11.391	69.49	10.910	70.6	12.221
78.59	10.160	78.99	11.947	78.92	11.841	79.9	12.950
88.06	11.521	88.30	12.803	88.44	13.773	89.3	14.411
94.00	12.703	94.59	13.712	94.77	15.470	95.5	16.211

## Data Availability

The original contributions presented in the study are included in the article; further inquiries can be directed to the corresponding author.
